# fMRI-based explanations for how meditation could modulate pain processing

**DOI:** 10.3389/fnins.2025.1561580

**Published:** 2025-05-16

**Authors:** Faly Golshan, Marla J. S. Mickleborough

**Affiliations:** Department of Psychology & Health Studies, University of Saskatchewan, Saskatoon, SK, Canada

**Keywords:** meditation, pain management, acute noxious pain, chronic pain, fMRI, open monitoring, focused attention

## Abstract

Meditation is a widely recognized umbrella term encompassing a diverse range of techniques with shared foundational characteristics, celebrated for their potential to alleviate mental and physical challenges. While subjective reports and behavioral studies have long highlighted meditation’s benefits, recent neuroscientific research has sought to provide tangible physiological evidence of its efficacy as a non-invasive intervention for managing physical pain. This review examines the neurophysiological mechanisms by which meditation influences brain activity in response to both acute and chronic pain experiences. Drawing on findings from functional magnetic resonance imaging (fMRI) studies, general models are categorized to explain how meditation alters cortical responses in both naïve and expert practitioners when exposed to pain stimuli. First, we discuss three major components of pain processing in the brain and analyze how meditation affects each stage. Next, we identify key brain regions consistently implicated in pain modulation through meditation, elucidating their roles in pain perception and regulation. Finally, we propose a framework for differentiating meditation techniques based on their distinct effects on pain experiences. These insights have significant implications for understanding the therapeutic potential of various meditation techniques for pain management, particularly in chronic conditions.

## 1 Introduction

Pain is a fundamental survival mechanism that protects organisms from threats by signaling disruptions in homeostatic balance. It is described as an unpleasant experience with sensory and emotional dimensions in response to actual or potential tissue damage (Merskey and Bogduk, 1994). The initiation, maintenance, perception and amplification of pain can be studied through various mechanisms, including psychosocial, movement system, and biological factors. While these mechanisms often overlap and occur simultaneously, each has distinct characteristics that contribute to the pain experience (Phillips and Clauw, 2011).

Biological factors influencing pain can be categorized into three primary mechanisms. First, nociceptive (peripheral) mechanisms arise from disruptions detected by the peripheral nervous system due to injury, or mechanical irritation. Some studies classify inflammatory pain as a subset of peripheral mechanisms (Chimenti et al., 2018), while others consider it as a distinct category (Xu and Yaksh, 2011). In this study, we categorize inflammatory mechanisms under nociceptive pain given their peripheral origin. Second, nociplastic pain mechanisms arise from non-nociceptive conditions resulting from an altered processing in the central nervous system (CNS) which can result from inflammation or central sensitization, a process characterized by heightened central excitability (Chimenti et al., 2018). Most chronic pain conditions, such a fibromyalgia and chronic migraine headache disorders, are examples of nociplastic pain mechanisms (Bułdyś et al., 2023). Finally, neuropathic pain mechanisms arise from lesions or diseases affecting the somatosensory system. This can occur such as when a nerve damage leads to pain, as seen in carpal tunnel syndrome, or when pain is a consequence of a metabolic condition such as diabetes (Phillips and Clauw, 2011).

During periods of pain, typically classified based on the biological characteristics of a noxious experience, the CNS triggers various emotional and cognitive responses. When pain becomes prolonged, individuals often engage in cycles of negative rumination and avoidance behaviors, which paradoxically intensify their pain experience (Zeidan et al., 2015). This highlights the importance of developing evidence-based strategies to help individuals regulate their behavioral and emotional responses to pain. Fostering conscious awareness through such strategies may help alleviate the perceived intensity of pain and prevents from the process of chronification.

One way to study pain responses in the CNS is through brain imaging techniques, such as functional magnetic resonance imaging (fMRI), which provide a spatial representation of cortical areas involved in sensory, cognitive, and psychological processing. fMRI measures changes in blood flow, offering high spatial resolution of functional connectivity and revealing activations and interactions among multiple brain regions during resting states or task-based conditions (Zeidan et al., 2015; Orme-Johnson et al., 1995; Gard et al., 2012).

A significant contribution of fMRI-based research is its role in refining the conceptualization of the “pain matrix,” a framework that challenges the traditional view of a single, centralized pain hub. Instead, it highlights a dynamic network of cortical and subcortical structures involved in pain processing (Garcia-Larrea and Peyron, 2013). Before exploring the connectivity patterns within this network, it is crucial for researchers to identify specific cortical and subcortical regions that have the potential to modulate pain. This understanding is particularly valuable for developing non-pharmaceutical interventions as alternative pain management strategies.

As for the frequency of pain across all different pain mechanisms, any type of pain that lasts less than 3 months is acute pain, which consistently activates certain cortical area during noxious stimuli (Jurisic et al., 2018). When pain persists for more than 3 months, it is classified as chronic pain, which is often associated with significant physiological and psychological complications and could involve with nociplastic mechanisms (Grichnik and Ferrante, 1991).

In order to alleviate pain, people can have different pharmaceutical and non-pharmaceutical alternatives, from applying a topical analgesic, taking abortive and preventative drugs, or even serotonin noradrenaline reuptake inhibitors in terms of chronic pain conditions (Chimenti et al., 2018). Recent studies indicating that prolonged use of abortive medicine to alleviate pain could itself cause the spark situations such as medication-overuse headaches (MOH) (Schwedt et al., 2022) has spurred interest in exploring non-pharmaceutical and complimentary interventions, such as meditation, alongside traditional pharmacological treatments to manage pain effectively.

Meditation encompasses a diverse range of mental training techniques that cultivate attention and heightened self-awareness to regulate both the mind and body (Hilton et al., 2017; Pozek et al., 2016). Currently, over 309 distinct meditation techniques exist, all sharing the common goals of enhancing self-regulation and expanding consciousness (Sedlmeier et al., 2012). Historically, meditation has demonstrated significant benefits for mental health and psychological well-being (Hilton et al., 2017; McGee, 2008). However, while its application in pain management has shown promising outcomes, neuroscientific evidence in this area is still evolving.

From a psychological perspective, meditation techniques engage multiple cognitive faculties, including attention, emotion regulation, reasoning, visualization, memory, and interoception (i.e., bodily awareness) (Shear, 2006) which could help with a modulated pain perception experience. The nature of these techniques varies, ranging from active to passive, effortless to disciplined exercises. Moreover, while attentional shifting is a fundamental aspect of meditation in healthy individuals, different techniques guide attention toward various focal points, such as thoughts, imagery, concepts, internal energy, bodily sensations, love, or spiritual entities (Sedlmeier et al., 2012). In pain conditions, individuals can learn to sustain their attention on or away from the painful stimulus based on the level of expertise and type of meditation.

Neuroscientific research commonly categorizes meditation techniques into two primary types (Sperduti et al., 2012; Lutz et al., 2008; Nash et al., 2013), particularly when studying its role in pain modulation (Tang et al., 2015): (1) Focused Attention (FA), which involves sustaining attention on a specific object while enhancing the ability to detect and disengage from distractions, and (2) Open Monitoring (OM), which fosters non-judgmental awareness of arising experiences within the mental continuum without fixating on a particular object (Deolindo et al., 2020). This dichotomy is well-supported in psychology and neuroscience (Shear, 2006; Goleman, 1988; Kristeller and Rikhye, 2008; Naranjo and Ornstein, 1971; Feuerstein, 2001; Walsh and Shapiro, 2006).

Despite their distinctions, FA and OM techniques are interrelated and share common neural mechanisms, involving brain regions such as the insula and precuneus (Wang et al., 2011). Consequently, some neuroscientific models conceptualize meditation as a spectrum, with concentrative meditation (emphasizing FA techniques) at one end and mindfulness meditation (predominantly OM-oriented) at the other (Cahn and Polich, 2006). Nonetheless, given the fundamental role of attention in meditation, the FA-OM dichotomy remains a widely accepted framework for classifying meditation techniques.

A defining characteristic of FA techniques is the use of an anchor, such as a mental image, breathing rhythm, or mantra, to maintain focus while inhibiting the processing of extraneous mental or environmental stimuli. These techniques are foundational in many traditional and spiritual practices, including Transcendental Meditation (TM) (Sedlmeier et al., 2012). In contrast, OM techniques cultivate deep awareness of thoughts, emotions, and bodily sensations without triggering reactive responses in the form of secondary thoughts or emotions. Notable examples of OM techniques include Vipassana, i.e., a Theravāda Buddhist meditation practice, and Mindfulness-Based Stress Reduction (MBSR), developed by Kabat-Zinn (1994), both of which aim to enhance individuals’ ability to monitor and regulate their physical and emotional states particularly during pain.

The application of meditation techniques as a cost-effective, accessible, and side-effect-free alternative for pain management is promising (Pozek et al., 2016; la Cour and Petersen, 2015). Early research on meditation and pain primarily relied on behavioral and clinical evidence, including subjective self-reports (Gard et al., 2012), which were susceptible to response biases. Additionally, randomized controlled trials (RCTs) have also traditionally depended on participants’ self-reported pain experiences during or after meditation practices (la Cour and Petersen, 2015; Hoge et al., 2013; Lin et al., 2022).

More recently, neuroscientific exploration using brain imaging has provided an understanding of how meditation influences cortical processing, further validating its efficacy in pain management. Brain imaging studies have identified distinct patterns of cortical activity and connectivity, which illustrate how meditation alters neural function in healthy individuals (Tang et al., 2015).

A meta-analysis of over 10 studies highlighted three key brain regions involved in attentional disengagement from irrelevant stimuli: the caudate, entorhinal cortex, and medial prefrontal cortex (Sperduti et al., 2012). However, concerns remain regarding the generalizability of these findings, as the studies included in the meta-analysis primarily examined traditional meditation styles mostly across expert individuals. More broadly, evidence suggests that OM techniques are associated with the activation of regions such as the anterior cingulate cortex (ACC), prefrontal cortex (PFC), insula, and striatum, while they are also linked to the deactivation of the posterior cingulate cortex (PCC) and amygdala (Tang et al., 2015).

Despite advancements in understanding the cortical mechanisms underlying OM techniques, much remains to be explored regarding how meditation practices across OM and FA categories differentially affect brain regions involved in various pain mechanisms—particularly in distinguishing between responses to induced pain in healthy subjects and chronic pain conditions. The primary objective is to identify the brain regions consistently involved across all types of pains conditions, whether acute or chronic, during meditation. Moreover, we aim to determine which meditation category, OM or FA, is more effective in managing chronic pain, given its greater complexity and impact on sufferers.

In this perspective article, we selected large-scale, gold standard studies that emphasize fMRI evidence of cortical activity associated with meditation during pain-induced and chronic pain conditions (Table 1). These studies serve as the foundation for our proposed perspective, which differentiates between OM and FA techniques in their application to various pain conditions.

**Table 1:** Description of previous literature on fMRI-based studies of pain modulation via meditation techniques.

Articles	Key highlights	Significant brain regions	Pain stimuli	Type of meditation	Participants	fMRI Indications	Focused attention or open monitoring?	Default mode network (DMN) or Salience network (SN)
Aytur et al. (2021)	improved functional connectivity across networks (DMN, SN, and FPN) correlate with behavioral outcomes, such as reduced depression, lower pain interference, and increased social participation.	DMN: mPFC, PCC, AG. SN: AI, ACC. FPN: dlPFC, IPL	Chronic musculoskeletal pain	4-week of ACT(two sessions of 90 min per week)	9 adult women with chronic musculosceletal pain	DMN: Decreased connectivity in mPFC and PCC (reflecting self-focused rumination and present-focused attention). SN: changes in AI, and ACC (suggesting reduced emotional reactivity and improved emotional regulation). Reduced coupling between DMN and SN indicate overlap between self-referential thinking and emotional processing of pain.	OM	DMN, SN
Berry et al. (2020)	Changes in both SN and DMN are associated with self-referential processing, salience detection and emotion regulation.	vPCC, TPJ, and dlPFC	Chronic low back pain	8-h self-compassion training	22 participants with low back pain.	Changes in vPCC activation (reduced emotional distress associated with pain), and TPJ (a shift in perceiving pain in relation to the self). Increased activation of dlPFC (better regulation of emotional response to pain).	OM	DMN, SN
Riegner et al. (2023)	The decoupling of the thalamus from the DMN interrupts connection between sensory pain signals and the self-referential processing that intensifies pain perception.	Thalamus, ACC, Insula, S1	Induced thermal pain	Mindfulness Meditation	40 healthy participants; 20 in mindfulness group and 20 in controlled audiobook listening group.	Improved functional connectivity across networks (DMN, and SN) correlate with behavioral outcomes, such as reduced depression, lower pain interference, and increased social participation.	OM	DMN
Kober et al. (2019)	Altered activity of DMN promotes a more detached, less self-focused processing of pain and reduced activation of SN helps decreased emotional salience, and intensity of pain via neutral and nonjudgmental experience of sensation.	ACC, PFC, insula, S1, dlPFC	Induced thermal pain	Mindfulness Meditation	18 healthy naïve meditators	Decreased activity in ACC (reduction in emotional distress), altered activation in insula (less emotional response to pain), activation of dlPFC (help with down-regulation or negative emotions.)	OM	DMN, SN
Hunt et al. (2023)	Focusing Mesocorticolimbic System Functional Connectivity. Greater vmPFC-rNAcc connectivity as a result of MSBR showed greater reductions in headache frequency. MBSR could improve pain catastrophizing, but headache severity could still remain the same.	rNAcc, vmPFC	Migraine headache pain	12-week MBSR training	98 participants with migraine headaches (50 receiving MBSR and 48 receiving stress management for headaches)	MBSR was associated with significant seed-to-voxel functional connectivity between rNAcc and vmPFC.	OM	SN
Fedeli et al. (2024)	mindfulness caused improved SN-insula connectivity was correlated with reductions in depression scores as a function of mindfulness.	Left PI, S1, ACC	Chronic Medication Overuse Headache (MOH)	Mindfulness Meditation (added to treatment as usual)	34 participants with chronic medication overuse headaches (17 mindfulness plus treatment and 17 treatment only)	Increased connectivity between SN and the lest posteriorior insula and sensorymotor cortex (causing pain modulation and body awareness). Increased cortical thickness in ACC and the insular cortex (emotion regulation, and pain perception).	OM	DMN, SN
Chen et al. (2023)	MBSR has the potential to influence brain structure and activity related to chronic pain and emotional processing.	AG, MFG, PHG, left AI and PI, SFGmed, SPL, postCG	Chronic pain	8-week MBSR	67 participants with chronic pain received MBSR (*n* = 40);and treatment as usual (*n* = 27)	MBSR was associated with increase in GMV, decreased amplitude of low-frequency fluctuations, and increased regional homogeneity.	OM	NA
Hatchard et al. (2022)	The control group of this study showed a decreased GMV of PHG, precuneus, MFG and right cuneus; probably due to the chronic pain experience.	AG, MFG, rPHG, right cuneus	Chronic neuropathic pain	8-week MBSR	23 participants with chronic pain:10 MBSR trainees and 10 waitlisted control	MBSR caused increased GMV in angular gyrus, middle frontal gyrus, right parahippocampal gyrus.	OM	NA
Smith et al. (2021)	The reduced functional connectivity may be related to both the descending motor and pain pathways.	S1, precuneus, dlPFC	Chronic neuropathic pain	8-week MBSR	23 participants with chronic pain:10 MBSR trainees and 10 waitlisted control	Reduced BOLD activity in somatosensory cortex, precuneus, and dorsolateral prefrontal cortex (correlated with decreased pain interference and improved emotional reactivity)	OM	DMN
Su et al. (2016)	Empathy, emotional awareness and their significant effect on attention switching	AIC, daMCC	Chronic pain	6-week MBSR: body scan, sitting meditation, Hatha yoga, walking meditation	34 participants: (18 pain afflicted and 16 controls with pain less than 1 score in McGill Pain Questionnaire)	Significant interaction between AIC and daMCC activity. A significant negative correlation between the results of “Short Form McGill Pain Questionnaire” and AIC-daMCC connection strength. Pain decreased significantly in pain-afflicted group but not in the control group after training, meditation may cause a significant change of attention to pain.	OM	SN
Orme-Johnson et al. (2006)	ACC showed the least significant change when associated with meditation.	Thalamus, ACC, PFC, central coordinates (mediolateral, anterior–posterior, and dorsoventral cortex)	Induced thermal pain	4-day Transcendental meditation (20 min daily)	24 participants (12 healthy controls and 12 long-term meditators with an average of 31 years of practice)	Long-term meditators show less cerebral blood flow in ACC, PFC, and thalamus in response to the painful stimulus. The changes brought by meditation is usually related to motivational-affective level than the sensory level.	FA	DMN, SN
Kakigi et al. (2005)	Professional practice of meditation may significantly modulate sensory processing of pain	Thalamus, S2-insula, ACC	Induced thermal pain	Yoga (abdominal breathing pattern)	One yoga master (38 years of experience)	Modulated activity of thalamus, SII-insula and cingulate cortex. The meditator claimed no sense of pain during meditation. Increased signal activities in frontal lobe, parietal lobe, midbrain increase during meditation	FA	SN
Grant et al. (2010)	The effect of meditation training levels on neural activations of pain	dlPFC, amygdala, MFG, hippocampus, mPFC/OFC, dACC, thalamus, insula	Induced thermal pain	Zen Meditation (Zazen)	13 professional meditators and 9 healthy controls	A negative correlation between pain-related activation and meditation experience. During the induced pain, there was a higher activation in dACC, thalamus, and insula in meditation group. Meditators showed a lower baseline pain sensitivity, no typical attention-related pain increases, and decreased sensory and affective components during mindful attention.	FA	SN
Zeidan et al. (2015)	A “gold standard” study comparing the analgesic mechanisms of mindfulness meditation with placebo and sham mindfulness	S2, ACC, putamen, dlPFC, hippocampus, OFC, ACC, IFG, MFG	Induced thermal pain	Mindfulness meditation (breathing technique, full flow of breath)	75 healthy naïve meditators	Reductions in DLPFC, mPFC, PCC. Greater activation in bilateral OFC, subgenual ACC, right anterior insula, and putamen. Pain intensity and pain unpleasantness reported to have the highest reduction in mindfulness meditation before and after the intervention when compared to other groups.	OM	SN, DMN
Kober et al. (2019)	No significant neural markers of PFC in response to negative emotion in meditating groups. Evidence showing that meditation acts differently from an emotion regulation strategy. The impact of meditation on Neural Pain Signature	Amygdala, PFC, ACC, thalamus	Induced thermal pain	Mindfulness meditation (breathing technique, full flow of breath)	17 naive meditators	Negative images: the activity in right amygdala reduced in accepting cue, pretty similar between neutral and negative images. Pain: daCC, medial frontal gyrus, sensorimotor regions: precentral and postcentral gyri, pre-SMA, anterior and posterior insula, thalamus, and cerebellum.	OM	SN
Zeidan et al. (2011)	Direct relation between meditation-related activation of some brain regions with pain modulation	ACC, AI, OFC	Induced thermal pain	Mindfulness meditation (attention to breath)	18 healthy subjects	Additional activation in ventral striatum, ventrolateral PFC, and amygdala (Meditation is different from a relaxing mode). changes in pain intensity and pain unpleasantness ratings	OM	SN
Lutz et al. (2013)	Expert meditation practice enhances neural mechanisms associated with attentional focus and emotion regulation during pain.	AI, aMCC	Induced thermal pain	Open Presence meditation (OM) compared to FA	14 long-term meditators (10,000 h of practice) and 14 novice controls	Neural habituation in aMCC. Meditation expertise can decrease the baseline activity prior to pain. Expert meditation practice-anticipation of a safe condition reduces activity in AI.	OM	SN
Gard et al. (2012)	Staying focused on the pain inducer and pain sensation. Top-down modulation of pain and decreased activation of brain regions involved in pain (e.g., insula/S2)	rACC, PFC, PI, S2, thalamus	Induced pain with an electrical stimulator	Mindfulness (Vipassana meditation)	34 (17) meditators and 17 controls	Reduced unpleasantness (22%) and anxiety (29%). Decreased activation of IPFC and increased activation in posterior insula during pain stimulation and increased activity in rACC during anticipation of pain	OM	NA

ACT = acceptance and commitment therapy; IFG = inferior frontal gyrus; rACC = rostral anterior cingulate cortex; AG = angular gyrus; IPL = inferior parietal lobe; rNAcc = rostral nucleus accumbens; AI = anterior insula; MBSR = mindfulness-based stress reduction; rPHG = right parahippocampal gyrus; ACC = anterior cingulate cortex; MFG = middle frontal gyrus; S1 = primary somatosensory cortex; aMCC = anterior midcingulate cortex; mPFC = medial prefrontal cortex; S2 = secondary somatosensory cortex; BOLD = blood oxygen level dependent; OM = open monitoring; SFGmed = superior medial frontal gyrus; DMN = default mode network; OFC = orbitofrontal cortex; SN = salience network; dACC = dorsal anterior cingulate cortex; PI = posterior insula; SMA = supplementary motor area; daMCC = dorsal anterior midcingulate cortex; PHG = parahippocampal gyrus; SPL = right superior parietal lobule; dlPFC = dorsolateral prefrontal cortex; PCC = posterior cingulate cortex; TPJ = temporoparietal junction; FA = focused attention; PFC = prefrontal cortex; vPCC = ventral posterior cingulate cortex; FPN = frontoparietal network; postCG = post central gyrus; vmPFC = ventromedial prefrontal cortex GMV = gray matter volume.

We begin by discussing the “three-tiered hierarchical model” of pain processing, which provides a broad framework for understanding meditation’s influence on pain-related neural networks. We then highlight key brain regions from the “neurological pain signature” (NPS) model which prior research has consistently identified as being modulated by meditation.

## 2 Three-tiered hierarchical perspective of pain processing in meditation

The three-tiered hierarchical model is proposed to organize pain-related neural networks into three categories based on the shared levels of processing that may involve specific brain regions: sensory, affective, and cognitive perceptive networks (Garcia-Larrea and Peyron, 2013). This perspective over pain processing separates the affective and cognitive dimensions due to their differing levels of consciousness and emotional influence; this is unlike some previous models which keep a dualistic approach by merging affective and cognitive components under one category (Garland et al., 2017).

### 2.1 Sensory-discriminative (nociception) network

Sensory network is responsible for processing signals transmitted from the spinal cord to the posterior thalamus before they reach conscious awareness. It plays a fundamental role in encoding the sensory aspects of pain, including its intensity, location, and duration. A key focus in pain research is determining whether specific techniques, such as meditation, can modulate this foundational level of pain perception. Investigating the extent to which meditation influences this network is essential for understanding its potential as an intervention for pain management. The primary brain regions involved in the sensory processing network of pain include the thalamus, which serves as a central relay for nociceptive signals (Habig et al., 2023), the posterior insula, which contributes to interoceptive awareness of pain (Bastuji et al., 2018), the cerebellum, which has been increasingly recognized for its role in pain sensation and modulation (Li et al., 2024), and both the primary and secondary somatosensory cortices, which encode detailed sensory characteristics of pain (Huang et al., 2020).

In fact, research on effects of meditation on this network has yielded mixed results. While some studies have found no changes in sensory pain processing due to OM practice, (Orme-Johnson et al., 2006; Mills and Farrow, 1981), others, such as Brown and Jones (2010), reported a significant attenuated activation in the secondary somatosensory cortex (S2), posterior insula and the thalamus. Similarly, Grant et al. (2010) demonstrated that Zen meditation can alter the activation of brain regions involved in the sensory encoding of a noxious pain stimulus in regions such as the insula, S2, and the thalamus. In alignment with these findings, Kober et al. (2019) observed reduced activity in sensorimotor regions such as postcentral gyri, pre-supplementary motor area (pre-SMA), posterior insula, the thalamus, and cerebellum, following the states of mindfulness and acceptance, a form of OM meditation. In relation to this network, meditation appears to modulate the perceived sensory properties of pain, yet more information is required to realize the degree of this impact. Despite having found a wider alteration in the sensory network through OM techniques (Brown and Jones, 2010; Kober et al., 2019), the contribution across all fMRI based studies on FA was the modulation of this system during pain (Orme-Johnson et al., 2006; Kober et al., 2019; Kakigi et al., 2005).

### 2.2 Affective-reappraisive (emotional) network

This category encompasses emotional arousal and escape responses to pain and is found to be altered across both FA and OM techniques in painful conditions. While there is major overlap in cortical regions between this network and the cognitive network involved in pain processing, distinguishing between salience processing and attention-based networks is crucial in meditation studies. The affective-reappraisal network is primarily responsible for the rapid, impulsive evaluation of pain based on previously learned experiences and emotional associations. This network plays a key role in shaping an individual’s immediate reaction to pain, often triggering automatic avoidance behaviors or heightened emotional responses. However, when constructively activated, it can facilitate a more adaptive, motivational, and reappraisal-based processing of pain (Seno et al., 2018). This shift allows individuals to reinterpret their pain experience, fostering greater self-empathy and resilience. Over time, such reappraisal mechanisms can contribute to improved emotion regulation, reducing the distress associated with pain and enhancing overall psychological well-being (Naor and Rohr, 2020).

The main brain regions involved in this network are ACC (Huang et al., 2020), anterior insula (Bastuji et al., 2018), and the amygdala (Seno et al., 2018). Studies suggest that the affective/motivational dimension of pain perception is often more impacted by meditation than the sensory dimension (Singer et al., 2004; Villemure and Bushnell, 2002). In the context of meditation techniques, this dimension is variably influenced and modified during pain processing. On one hand, increased activation in brain regions such as the ACC and anterior insula has been linked to reducing pain expectations and anticipation during meditation (Brown and Jones, 2010). On the other hand, some studies report a significant decrease in the activity of emotion-related brain networks such as the connection between the amygdala with hippocampus which plays an important role in associating contextual learning about previous experiences of pain (Garcia-Larrea and Peyron, 2013; Garland et al., 2017).

Evidently, the affective-motivational aspect of meditation plays a crucial role in regulating empathy; the same network which is central to managing stress, anxiety, and pain-related distress. Early pain sensations often trigger negative appraisals by associating current experiences with past pain memories, which amplifies distress. Meditation appears to weaken this association, enabling individuals to disengage from pain catastrophizing and approach pain with reduced emotional reactivity (Zeidan et al., 2015).

### 2.3 Cognitive-evaluative (attentional) network

This network which shares some brain areas with the reappraisal-motivational network (Garcia-Larrea and Peyron, 2013) and is sometimes grouped together with it due to overlapping functions, despite the cognitive-perceptive network’s engagement in higher-order processes. During pain experiences, attentional processing of noxious stimuli and orientation toward the pain source can significantly alter the perception of pain, meaning that anticipating pain can enhance synchrony among brain regions encoding the sensory properties of noxious stimuli (Ohara et al., 2006).

Studies have shown that individuals with a heightened fear of pain exhibit attentional biases toward potential threats, often specific to their condition (Koster et al., 2006; Vago and Nakamura, 2011; Okifuji and Turk, 2016). While attention orienting is a core component of meditation, most meditation techniques go far beyond a simple redirection of attention (Zeidan et al., 2015). Instead, they can significantly reduce emotional responses and catastrophizing behaviors by flexibly orienting attention toward or away from pain, depending on the practice. It seems like mindfulness techniques such as decentering involve actively observing the source of physical pain, redirecting attention to the emotions elicited by the pain, and ultimately maintaining awareness of the pain from a detached perspective (Kober et al., 2019).

Meditation techniques differ widely in their approach to attentional practice (Grant et al., 2010). Yet what remains the same is that all meditation techniques contribute to an attentional shift to/from the source of pain. Meditation also enhances inhibitory control, helping individuals dissociate current pain from previous painful experiences (Lutz et al., 2013). Rather than being trapped in the pain sensation, meditation enables a flexible and fluid transformation of attention and responses.

As could be seen, evidence supports that meditation influences all categories of the three-tiered hierarchical model of pain processing. Nevertheless, the concern with this model is regarding the overlapping cortical regions which make it hard to distinguish if meditation is mostly helpful at either the sensory, emotional or the cognitive level. Therefore, we further discuss meditation experience from another perspective which mainly focuses on pinpointing most mentioned regions in literature reviews.

## 3 Neurological pain signature (NPS) of brain areas involved in meditation

The NPS is an objective, multivariate neural pattern that encompasses brain regions consistently activated during nociception and pain processing (Wager et al., 2013). By comparing individual neural data against the NPS, the intensity of pain can be reliably predicted, offering a valuable tool for objectively assessing pain experiences. The main role of NPS has been to learn about efficacy of analgesic treatments (Kober et al., 2019; Wager et al., 2013; López-Solà et al., 2022), but such a signature can also elaborate more on non-pharmaceutical techniques, such as meditation, to ensure if a technique is appropriately implemented and whether it is functional for subjects or not.

Given the importance of reaching NPS model for meditation techniques, here we specify some fMRI evidence of the most frequently mentioned brain regions in both pain-induced and chronic pain conditions encouraging future studies to elaborate on the connectivity of such areas with each other.

### 3.1 Dorsolateral prefrontal cortex (DLPFC)

Dorsolateral prefrontal cortex is a frequently cited brain region in fMRI studies investigating pain and meditation experiences (Zeidan et al., 2015; Grant et al., 2010; Kober et al., 2019; Berry et al., 2020; Smith et al., 2021). It plays a critical role in moderating pain signals and contributes to a more regulated perception of pain. Specifically, research indicates that the DLPFC acts as a key player in bridging cognitive and emotional regulation in individuals experiencing pain, particularly as influenced by OM techniques (Berry et al., 2020; Smith et al., 2021).

A central function of DLPFC is its role in empowering non-judgmental awareness of sensations, a hallmark of OM techniques (Lutz et al., 2013). Studies have demonstrated that this region exhibits increased activation during meditation for chronic pain (Berry et al., 2020; Kober et al., 2019). Considering the literature demonstrating an association between heightened activation of the DLPFC in chronic musculoskeletal and low back pain, it can be inferred that this region plays a crucial role in the top-down modulation of pain through mechanisms such as neuroplasticity, nociception, and neuropathy. The activation of the DLPFC also reflects its involvement in working memory, a function that is particularly relevant to OM meditation. In contrast, during acute pain among healthy controls, DLPFC’s activation appears to be decreased instead when participants were doing OM (Grant et al., 2010). Such a deactivation in this region could be associated with a shift away from the cognitive effort toward effortless pain modulation, and therefore a reduced pain perception. While these studies primarily focus on OM strategies, the observed changes in DLPFC activation may be associated with the type of pain, whether induced or chronic.

### 3.2 Anterior cingulate cortex (ACC)

Anterior cingulate cortex plays a pivotal role in both pain processing and mindfulness, serving as a key hub for emotional and cognitive regulation (Tang et al., 2015; Zeidan et al., 2011). Based on our review, ACC is one of the most frequently cited regions associated with interpreting unpleasant states and emotional distress linked to pain in both meditation categories and across induced pain and chronic pain conditions. It ultimately regulates pain perception through various cognitive strategies (Zeidan et al., 2015; Gard et al., 2012; Orme-Johnson et al., 2006; Kober et al., 2019; Zeidan et al., 2011; Fedeli et al., 2024; Riegner et al., 2023; Aytur et al., 2021; Zeidan et al., 2011; Chen et al., 2023).

Meditation practices target ACC to enhance its functions, including sustaining attention on the present moment, detecting discrepancies between actions and expected outcomes in critical conditions, and, most importantly, regulating emotion by supporting metacognition and heightened self-awareness during painful situations. This suggests that ACC contributes to fostering a positive attitude and acceptance toward impending stimuli, which may enhance coping strategies during pain.

Anterior cingulate cortex is commonly implicated in OM practices (Zeidan et al., 2015; Kober et al., 2019; Zeidan et al., 2011; Fedeli et al., 2024; Riegner et al., 2023; Aytur et al., 2021; Zeidan et al., 2011; Chen et al., 2023), although it is also found to play a role in FA techniques (Xu and Yaksh, 2011; Nash et al., 2013). Most studies reporting ACC activation involve healthy subjects receiving an induced pain (Zeidan et al., 2015; Gard et al., 2012; Orme-Johnson et al., 2006; Grant et al., 2010; Kober et al., 2019; Zeidan et al., 2011; Riegner et al., 2023). Compared to the DLPFC, fewer studies have linked ACC modulation to improved chronic pain outcomes through meditation (Fedeli et al., 2024; Aytur et al., 2021). Fadeli et al.’s study on chronic migraine and medication-overuse headaches (MOH) found increased cortical thickness in the ACC among individuals with MOH compared to healthy controls (Fedeli et al., 2024). Another study on chronic musculoskeletal pain suggests that the salience network is diminished, as indicated by reduced activity in the ACC along with the primary somatosensory cortex (S1). Although the existing literature does not allow for definitive conclusions across all pain mechanisms, the ACC serves as a hub for embedding an automatic state of non-reactivity to pain stimuli. It is more closely associated with nociceptive pain mechanisms, as observed in studies of induced pain in healthy controls. In general, it can be inferred that meditation practices, particularly OM techniques, modulate ACC activity by reframing pain as a neutral sensory experience through constructive cognitive strategies.

### 3.3 Insula

The insula is another frequently cited brain region in fMRI studies (Zeidan et al., 2015; Gard et al., 2012; Grant et al., 2010; Kober et al., 2019; Kakigi et al., 2005; Fedeli et al., 2024; Riegner et al., 2023; Aytur et al., 2021; Zeidan et al., 2011; Chen et al., 2023; Nakata et al., 2014) and is involved in enhancing interoceptive awareness, moderating emotional reactivity, and improving cognitive control. This region is one of the key hubs for bridging the sensory and emotional processing of pain to each other with the posterior insula receiving direct input from spinothalamic pathway and evaluating pain intensity, location and sensory recognition of a noxious stimulus and connecting it to the anterior insula for more emotional processing and connecting the information to the rest of the limbic system (Bastuji et al., 2018).

Chronic pain conditions are often associated with hyperactivation of the insula, reflecting increased maladaptive interoceptive awareness of pain indicators, which can lead to heightened emotional distress in individuals experiencing pain (Zeidan et al., 2015; Zeidan et al., 2011).

Evidence suggests that meditation can increase insula activation in response to acute pain (Zeidan et al., 2015; Grant et al., 2010). However, in chronic pain, meditation generally moderates emotional responses through the modulation of insula activity (Fedeli et al., 2024; Aytur et al., 2021; Chen et al., 2023). Additionally, the involvement of the insula in meditation techniques is not limited to OM strategies; FA practices have also been shown to modulate insula activity as well (Grant et al., 2010; Kakigi et al., 2005).

Overall, meditation appears to regulate insular activation, helping to control hypervigilance and catastrophizing responses to pain. The key factor is not just the activation of this region but also its connectivity within the salience network (SN), particularly with the ACC (Fedeli et al., 2024; Aytur et al., 2021). This regulation is crucial, as it may help delay the chronification process associated with prolonged negative emotional responses across different pain mechanisms.

### 3.4 Thalamus

The thalamus is known for its role in mediating and integrating different forms of somatosensory, noxious, and vestibular stimuli (Habig et al., 2023). As a relay station for the sensory perception of pain, the thalamus plays a critical role in mediating pain relief through meditation practice specifically when a mechanical pain is induced. In the thalamus, critical sensory information, including nociceptive signals, is processed before reaching other areas of the cortex. fMRI evidence demonstrates decreased thalamic activity during meditation, suggesting that meditation effectively filters nociceptive information and modulates the sensory transmission of pain signals to higher cortical regions (Gard et al., 2012; Orme-Johnson et al., 2006; Grant et al., 2010; Kober et al., 2019; Kakigi et al., 2005; Riegner et al., 2023).

Changes in thalamic activity is often observed in professional meditators. For example, the thalamus was found a kay factor in a study observing a yoga master during practice (Kakigi et al., 2005), a result consistent across various meditation techniques (Gard et al., 2012; Grant et al., 2010). This highlights meditation’s impact, which extends beyond modulating the affective aspects of pain to directly influencing sensory processing, particularly at professional levels of practice. Research shows that both FA practices (Orme-Johnson et al., 2006; Grant et al., 2010; Kakigi et al., 2005) and OM techniques (Gard et al., 2012; Kober et al., 2019; Riegner et al., 2023) have a comparable impact on thalamic modulation; yet FA practices highlight this area as critically impacted region during practice (López-Solà et al., 2022).

In general, the thalamus is one of the initial regions where sensory pain signals and nociceptive inputs to higher emotional and cognitive levels can be decoupled (Riegner et al., 2023) and evidence supports the assertion that long practice of meditation has a clear impact on sensory modulation, as demonstrated by mediated activation in areas such as the thalamus and cerebellum in response to noxious stimuli (Kober et al., 2019; Nakata et al., 2014). However, there is currently no evidence to suggest that meditation modulates thalamic activation in chronic pain conditions. This suggests that meditation, particularly OM, may be more effective at addressing the emotional and cognitive dimensions of pain rather than its sensory aspects in individuals with chronic pain.

## 4 Discussion

**Figure 1:**
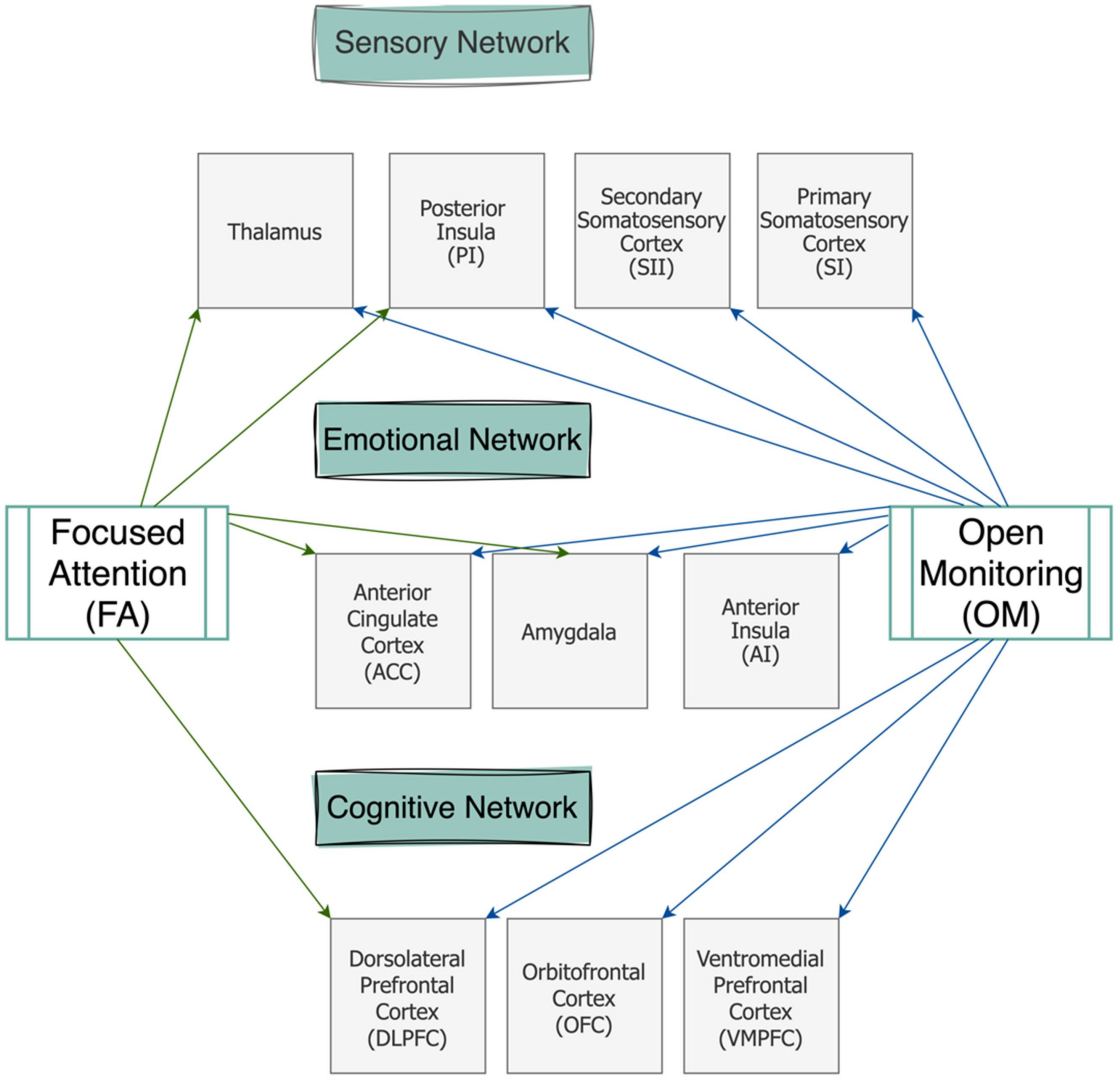
Schematic representation of the brain regions involved in the three-tiered networks of pain modulation during two meditation categories (FA and OM).

We discussed how fMRI studies support a categorical theory of pain (i.e., three-tiered hierarchical perspective of pain) and pinpointed some common regions which could inspire further studies on a holistic model of pain (i.e., NPS) during meditation. Figure 1 elaborates on cortical regions at each of the three-tiered networks in association with OM and FA techniques. Here we provide a practical perspective for meditation’s efficacy in pain management discussing which category of meditation may be more suitable for addressing chronic pain conditions.

### 4.1 Meditation practice beyond placebo effect

The fMRI studies show that meditation can alleviate pain through multiple mechanisms. It promotes self-compassion and grounding in the present moment, reducing pain-related stress and emotional discomfort (Su et al., 2016; Okifuji and Turk, 2016). Additionally, meditation may attenuate physiological reactions to pain-related distress, potentially by increasing the secretion of endogenous endorphins (Orme-Johnson et al., 2006). Meditation techniques are also associated with key hypometabolic changes, such as lower respiration and heart rates, which significantly influence the pain experience (Beary and Benson, 1974). An ongoing debate concerns whether meditation operates distinctly from placebo effects.

Some studies attribute its benefits to factors such as facilitator attention, body posture, practice characteristics, and the environment (Zeidan et al., 2015; Tang et al., 2015; Zeidan et al., 2012). Notably, DLPFC plays a role in both meditation and placebo analgesia, potentially explaining why placebo effects can similarly reduce perceived pain (Wager and Atlas, 2015). However, a gold-standard fMRI study by Zeidan et al. (2015) found that reduced DLPFC activity during mindfulness meditation differs significantly from placebo and sham meditation groups. Behavioral reports from this study, such as decreased pain unpleasantness and intensity in the meditation group, further support meditation’s unique, non-placebo benefits.

### 4.2 Practical implications of meditation for chronic pain

**Figure 2:**
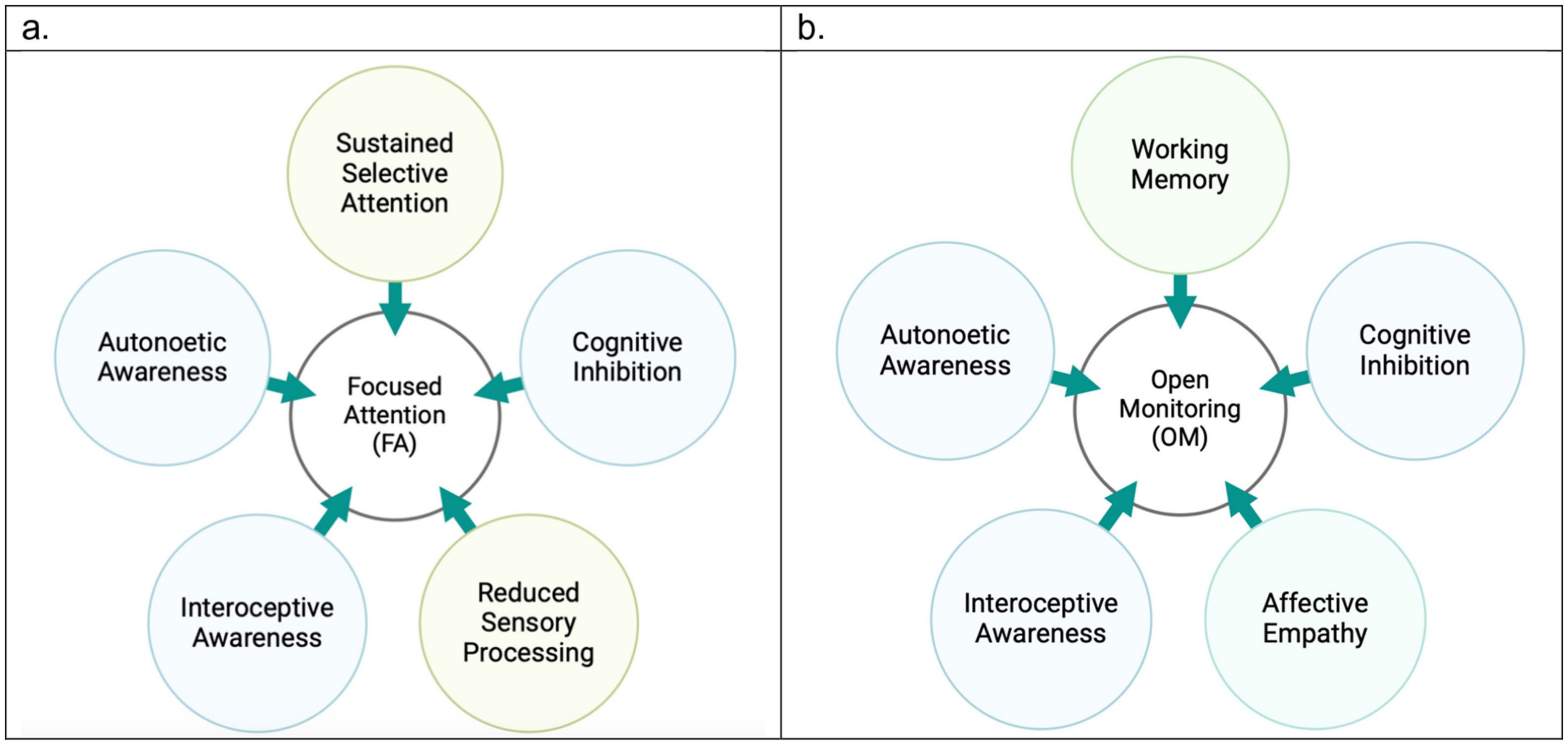
Major factors contributing to pain modulation in the two meditation categories.

Knowing that pain mechanisms are different from one another, here we explained how meditation techniques have been found helpful for three categories of nociceptive, neuroplastic, and neuropathic pain. A key question here is which meditation technique is most effective for pain management of different pain mechanisms. Previous research supports our perspective that FA and OM categories act distinctively in addressing acute or chronic pain conditions. Building on the three-tiered hierarchical perspective of pain, we differentiate between FA and OM meditation techniques in pain management. Figure 2 highlights the key factors associated with each of the techniques during pain modulation particularly by emphasizing that while OM is more potential to engage working memory, FA requires sustained attention on a task, so it reduces sensory processing of pain. We also assert that FA techniques require greater levels of expertise and are particularly effective for acute pain. In contrast, OM techniques are more accessible and beginner-friendly practices that foster a broader, non-reactive awareness of pain, making it especially beneficial for chronic pain conditions. We underscore that while FA primarily modulates sensory and emotional responses, OM engages a more comprehensive framework, integrating emotional, cognitive, and sensory regulation.

Based on the previous research, OM practices engage a wide range of cortical regions, including the superior temporal gyrus, superior parietal lobule, inferior frontal gyrus, precuneus, OFC, transverse temporal gyrus, fusiform gyrus, parahippocampus, and amygdala (Zeidan et al., 2015; Aytur et al., 2021; Zeidan et al., 2011; Manna et al., 2010). Behavioral studies indicate that naïve OM practitioners report reduced pain unpleasantness without significant changes in pain intensity (Perlman et al., 2010), suggesting a stronger link to affective network activation, particularly within the limbic system (Nakata et al., 2014).

In contrast, FA predominantly activates regions such as the ACC, and the thalamus (Wang et al., 2011; Orme-Johnson et al., 2006; Grant et al., 2010; Kakigi et al., 2005). However, fMRI evidence implies that FA’s efficacy is more pronounced in long-term or expert meditators (Orme-Johnson et al., 2006; Grant et al., 2010; Kakigi et al., 2005; Manna et al., 2010). While FA may mediate sensory aspects of pain perception along with affective and cognitive levels, its generalizability for chronic pain remains uncertain due to a lack of fMRI-based studies on FA practices in chronic pain conditions.

**Figure 3:**
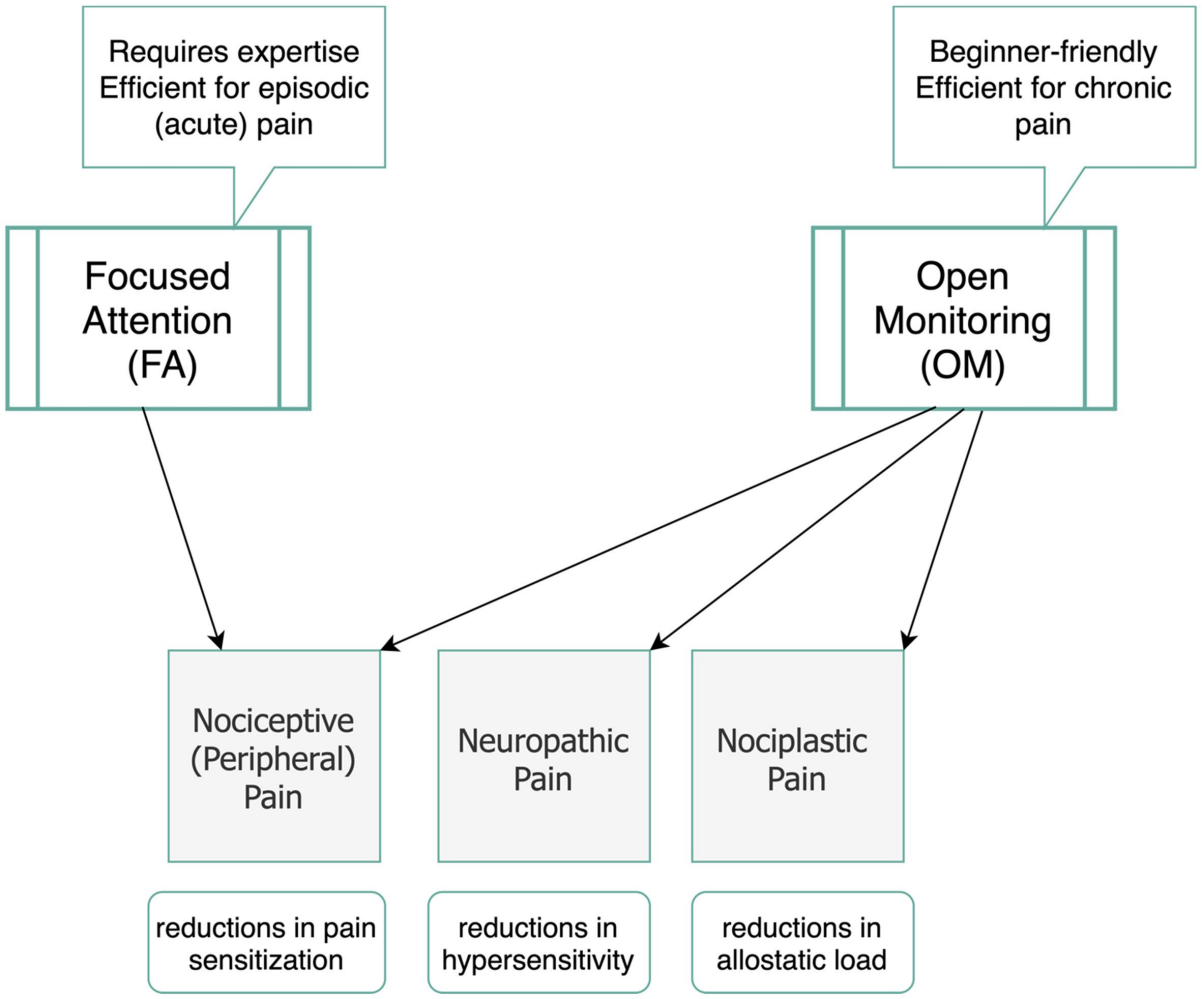
Suggested meditation categories (OM and FA) and their potential efficacy in modulating pain mechanisms.

Behavioral comparisons between OM and FA suggest that OM is more effective in reducing pain unpleasantness, even without altering pain intensity (Villemure and Bushnell, 2002). This makes OM techniques particularly suitable for naïve meditators seeking pain management, as they may more effectively promote acceptance and coping strategies in individuals with little or no prior meditation experience compared to FA techniques (Perlman et al., 2010; Ochsner et al., 2012). Additionally, OM helps individuals remain present in their experience of chronic pain by reducing hippocampal activity, which diminishes the mental and emotional processing of short-term pain into long-term memory (Hunt et al., 2023; Hatchard et al., 2022). This reduction in hippocampal activity enables individuals to moderate their behavioral reactivity by learning to dissociate their lived experience of pain from their current state, thereby reducing tendencies toward catastrophizing or hypervigilance about future pain. Figures 3 displays a summary of our perspective regarding the efficacy of OM and FA techniques for different pain mechanisms and different conditions of acute and chronic pain.

In sum, fMRI evidence provides compelling support for the efficacy of meditation as a valuable non-pharmaceutical alternative for managing pain, especially in chronic conditions. While this review has identified distinct patterns of brain activation associated with various meditation techniques, it also underscores the need for further research to explore these differences in greater detail. For instance, future studies could focus on delineating how FA and OM differentially activate brain regions and networks involved in pain perception and regulation. Such investigations would not only enhance our understanding of the neural mechanisms underpinning meditation’s efficacy but also help identify optimal meditation techniques for specific pain conditions, tailoring interventions to maximize therapeutic outcomes. By bridging the gap between neuroscience and clinical practice, these advancements could further establish meditation as a cornerstone in the management of chronic pain and related disorders.

## 5 Conclusion

Meditation encompasses a spectrum of techniques, ranging from focused attention (FA) to open monitoring (OM) strategies, which foster an aware mental state that enables individuals to focus on the sensory aspects of a noxious stimulus while voluntarily modulating their cognitive processing and emotional reactivity to painful experiences. fMRI-based evidence highlights the multidimensional influence of meditation on the brain in mitigating pain. This review explored the impact of meditation on three key layers of pain processing; sensory, cognitive, and emotional, in both acute and chronic pain conditions, elaborating on the most implicated brain regions involved in pain management through meditation techniques. In this perspective article, we propose that the primary distinction between FA and OM strategies lies in the level of the practitioner’s proficiency. Most fMRI evidence for FA techniques has been derived from studies on experienced meditators undergoing acute pain induction. From a neuroscientific perspective, we suggest that OM may be a more practical and accessible technique for chronic pain management in naïve meditators, as it facilitates acceptance, reduces pain-related emotional distress, and promotes coping strategies even without extensive prior training.

## Data Availability

The original contributions presented in the study are included in the article/supplementary material, further inquiries can be directed to the corresponding author.
